# Comparative Assessment of miR-185-5p and miR-191-5p Expression: From Normal Endometrium to High-Grade Endometrial Cancer

**DOI:** 10.3390/cells13131099

**Published:** 2024-06-25

**Authors:** Sergio Antonio Oropeza-de Lara, Idalia Garza-Veloz, Bertha Berthaud-González, Tania Guillermina Tirado-Navarro, Reinaldo Gurrola-Carlos, Bernardo Bonilla-Rocha, Ivan Delgado-Enciso, Margarita L. Martinez-Fierro

**Affiliations:** 1 Molecular Medicine Laboratory, Academic Unit of Human Medicine and Health Sciences, Universidad Autonoma de Zacatecas, Carretera Zacatecas-Guadalajara Km 6 Ejido la Escondida, Zacatecas 98160, Mexico; sergioantoniooropeza@gmail.com (S.A.O.-d.L.); dra.tania.tirado@gmail.com (T.G.T.-N.); reinaldogc@gmail.com (R.G.-C.); bern.ave96@gmail.com (B.B.-R.); 2 Hospital General “Luz González Cosío”, Circuito el Orito, Cd. Administrativa, Zacatecas 98160, Mexico; bberthaud@hotmail.com; 3 Department of Molecular Medicine, School of Medicine, University of Colima, Av. Universidad No. 333, Las Viboras, Colima 28040, Mexico; ivan_delgado_enciso@ucol.mx; 4 Department of Research, Colima Cancerology State Institute, IMSS-Bienestar Colima, Colima 28085, Mexico

**Keywords:** miRs, biomarker, endometrial cancer, epi-miRNAs, oncomiRNAs, tumor suppressor miRNAs

## Abstract

Endometrial cancer (EC) is a significant cause of cancer-related deaths in women. MicroRNAs (miRs) play a role in cancer development, acting as oncogenes or tumor suppressors. This study evaluated the diagnostic potential of hsa-miR-185-5p and hsa-miR-191-5p in EC and their correlation with clinical and histopathological features. A cross-sectional study analyzed formalin-fixed, paraffin-embedded tissue samples from 59 patients: 18 with EC, 21 with endometrial hyperplasia (EH), 17 with normal endometrium (NE), and 3 with endometrial polyps (EPs). Quantitative reverse transcription-polymerase chain reaction and TaqMan probes were used for miR expression analysis. The Shapiro–Wilk test was used to analyze the normal distribution of the data. Subsequently, parametric or non-parametric tests were used to evaluate the associations between the expression levels of each miR and clinical parameters. Both miRs were underexpressed in some precursor and malignant lesions compared to certain NE subtypes and benign lesions. Specifically, hsa-miR-185-5p showed underexpression in grade 3 EC compared to some NE and EH subtypes (FC: −57.9 to −8.5, *p* < 0.05), and hsa-miR-191-5p was underexpressed in EH and EC compared to secretory endometrium and EPs (FC: −4.2 to −32.8, *p* < 0.05). *SETD1B*, *TJP1*, and *MSI1* were common predicted target genes. In conclusion, hsa-miR-185-5p and hsa-miR-191-5p are underexpressed in EC tissues, correlating with histopathological grades, highlighting their potential as diagnostic biomarkers and their role as tumor suppressors in EC.

## 1. Introduction

Endometrial cancer (EC) is the most commonly identified gynecologic malignancy among postmenopausal women in industrialized countries. According to the World Health Organization (WHO), in Mexico, there were 7266 cases of this disease in 2018, placing it below cervical cancer in the ranking of malignant neoplasms of the female genital tract [1]. Data from Cancer Statistics indicate that in 2024, there will be an estimated 67,880 new cases of EC, representing 3.3% of all new cancer cases in the United States of America (USA), with 13,250 deaths attributed to the disease, accounting for 2.1% of all cancer fatalities [2].

Micro-RNAs (miRs) are non-coding RNAs of 19–24 nucleotides that are responsible for regulating gene expression [3] by binding to the untranslated regions (UTRs) of target messenger RNA (mRNA) and inhibit translation by blocking mRNA transcription or causing mRNA degradation [4,5]. Each miR has the potential to regulate genes (typically about 500), whereas each gene is often the target of several different miRs [6]. Tumor suppressor miRs prevent tumor development by negatively regulating oncogenes, whereas oncogenic miRNAs (oncomiRs) often promote tumor development by negatively inhibiting tumor suppressors or genes that control apoptosis [7]. A family of miRs, the metastamiR, has been shown to have pro- and antimetastatic effects [8]. The epi-miRs are distinguished by their ability to directly or indirectly direct the expression of several epigenetic regulators such as DNA methyltransferases (DNMTs) and histone deacetylases (HDACs) [9,10]. This is believed to be how miRs are associated with the development of different types of cancer, such as colorectal, breast, ovarian, and EC [11,12,13,14].

MiR-185-5p and miR-191-5p have frequently been reported as being downregulated in various malignancies and considered to act as broad tumor suppressors [15,16,17,18,19,20]. However, to our knowledge, the expression of both miRs in EC has not been extensively studied. Since it has been observed that both miRs could function as biomarkers in other types of neoplasms, studying the expression of these miRs in this type of cancer could help elucidate their role and usefulness as biomarkers. Overexpression of miR-185-5p inhibits the polymerization of F-actin induced by S100A8/A9 and reverses epithelial–mesenchymal transition in breast cancer by modulating the receptor for advanced glycation end-products (RAGE) in vitro [15]. MiR-185 is a tumor suppressor because it inhibits cell proliferation and anchorage-independent growth in immunocompromised mice, making it a potential diagnostic biomarker in EC [21]. The miR-191 can regulate apoptosis, proliferation, migration, and invasiveness in renal, breast, and endometrial carcinoma cell lines, and it could thus be an important miR that modulates responses to apoptotic signals in EC [19,22,23]. Given the above data, we hypothesized that compared with non-malignant tissue samples, miR-185-5p and miR-191-5p are underexpressed in EC tissue samples and that the expression levels of both miRs may be associated with histopathological diagnosis. The aim of this study was to evaluate the usefulness of tissue expression of miR-185-5p and miR-191-5p as diagnostic markers of EC and their correlation with clinical and histopathological variables of the disease.

## 2. Materials and Methods

### 2.1. Patients and Samples

A cross-sectional case-control study was carried out. Fifty-nine formalin-fixed, paraffin-embedded (FFPE) tissue samples collected between 2017 and 2021 were obtained from the Surgical Pathology files of the Zacatecas General Hospital “Luz González Cosío”, in Mexico. The pathological diagnosis of the tissue samples was as follows: 18 of EC (17 endometrioid endometrial cancer [EEC] and 1 serous endometrial carcinoma [SEC] samples); 17 non-related normal endometrium samples (NE: 7 proliferative [PE] and 10 secretory endometrial [SE] samples), which were included as normal controls; 21 samples had a diagnosis of endometrial hyperplasia (EH: 16 endometrial hyperplasia without atypia [EHWA] and 5 endometrial hyperplasia with atypia/endometrial intraepithelial neoplasia [EHA/EIN] samples), and 3 endometrial polyp (EP) specimens. Inclusion criteria for each subgroup included the diagnosis only of NE, EP, EH, and EC, respectively. Exclusion criteria for all cases included the presence of any other type of neoplasm. The EC, EH and EP cases were reviewed and classified using the 2014 WHO criteria [24]. Tumors were staged according to the 2009 International Federation of Gynecology and Obstetrics (FIGO) guidelines [25]. The study was approved by the Research Ethics Committee of the Academic Unit of Human Medicine and Health Sciences CONBIOETICA-32-CEI-002-20220328, with the following registration number: CEI-UAMHyCS-02-2022. The recommendations of the STROBE guidelines were considered during the manuscript preparation.

### 2.2. Pathological Analyses and RNA Extraction

The slides stained with hematoxylin and eosin used for diagnosis, along with a new slide prepared from the paraffin block of each patient, were reviewed by an independent pathologist according to the criteria of the WHO Classification of Tumors of Female Reproductive Organs, fourth edition [24]. The purpose was to corroborate the histopathological diagnosis of each sample and verify the presence of at least 80% of target tissue according to each group. Total RNA samples were extracted from two 10 µm thick slices of the FFPE tissues using the miRNeasy FFPE Kit (Qiagen (Hilden, Germany), Cat. No. 217504), according to the manufacturer’s instructions. The quality and quantity of RNA were assessed using the NanoDrop 2000c spectrophotometer (Thermo Fisher Scientific, Wilmington, DE, USA). Samples with 260/280 > 1.7 and 260/230 > 1.8 ratios were used for further analysis.

### 2.3. Quantitative Real-Time Polymerase Chain Reaction

A total of 2 µL of total RNA (100–300 ng) was reverse transcribed into complementary DNA (cDNA) by the TaqMan Advanced miRNA cDNA Synthesis Kit (Applied Biosystems (Waltham, MA, USA), Cat. No. A28007). The kit produces cDNAs from mature miRNAs in the total RNA samples by extending the 3′ end of the mature transcript through poly (A) addition and then lengthening the 5′ end by adaptor ligation. The modified miRNAs then undergo universal reverse transcription followed by amplification to increase uniformly the amount of cDNA from all miRNAs. The prepared cDNA was diluted 1:10 in nuclease-free water. The single-tube TaqMan Advanced miRNA Assay (Applied Biosystems (Waltham, MA, USA), Cat. No. A25576) was used to quantify mature miRs on a quantitative real-time polymerase chain reaction (RT-qPCR) and the StepOnePlus Real-Time PCR system (Applied Biosystems, Waltham, MA, USA). The cycling conditions applied were the following: 95 °C for 20 s, 1 cycle (enzyme activation); 40 cycles including 95 °C for 1 s (denaturation), and 60 °C for 20 s (annealing/extension). The 10 μL PCR reaction mixture included 0.5 μL of RT product, 5 µL 2X TaqMan Fast Advanced Master Mix (Applied Biosystems (Waltham, MA, USA), Cat. No. 4444557), 0.125 µL of the primer and probe mix of the TaqMan Advanced miRNA Assay (20X) (Applied Biosystems (Waltham, MA, USA), Cat. No. A25576), and 4.37 μL of nuclease-free water (Integrated DNA Technologies, Inc., Coralville, IA, USA), Cat. No. 11-05-01-04). The general data of the miR assays used are as follows: (1) hsa-miR-185-5p (Applied Biosystems (Waltham, MA, USA), Assay ID: 477939_mir; Stem-loop Accession number: MI0000482; Mature miRNA Sequence: UGGAGAGAAAGGCAGUUCCUGA) and (2) hsa-miR-191-5p (Applied Biosystems (Waltham, MA, USA), Assay ID: 477952_mir; Stem-loop Accession number: MI0000465; Mature miRNA Sequence: CAACGGAAUCCCAAAAGCAGCUG). All the samples were run in duplicate. Analysis of relative gene expression data of each miR was calculated using the 2^−ΔΔCq^ method [26] and the RNU44 (Applied Biosystems (Waltham, MA, USA), Assay ID: 001094) as endogenous control.

### 2.4. Statistical Analysis

Continuous variables were presented as mean ± standard deviation or median (interquartile range), and categorical variables as frequencies and percentages. The Shapiro–Wilk test was used to assess normality, and variance was evaluated accordingly (Brown–Forsythe test). Subsequently, parametric or non-parametric tests (such as the Student’s *t*-test, one-way ANOVA, and/or Mann–Whitney U test) were used to analyze the associations between the variables. The correlation coefficients of miR-185-5p and miR-191-5p were calculated using the Pearson correlation test. Statistical analyses were performed using SigmaPlot version 14.5 software (Grafiti LLC, Palo Alto, CA, USA). The DIANA-mirPath v.3 and v.4 web server (University of Thessaly, Larissa, Greece) was used to identify the target genes and cell signaling pathways in which both miRs were involved. A *p*-value less than 0.05 was considered statistically significant. Also, heat maps were generated for both miRs where each histological subtype was analyzed. For this analysis, the fold change (FC) was calculated and used during comparisons. The clustering of both miRs across different endometrial lesions was performed using fold-change and visualized as heatmaps with the Heatmapper software (http://www.heatmapper.ca/) (University of Alberta, Edmonton, AB, Canada) [27]. An interactome was created using the STRING database v.12.0 (Swiss Institute of Bioinformatics, Lausanne, Switzerland) [28] centered on the TJP1 protein. To construct the interactome, we queried the STRING database with the TJP1 protein identifier, filtering interactions based on a high confidence score (>0.7). The resulting data were used to build a protein–protein interaction network centered on TJP1. From this network, the top 10 most highly interacting proteins were identified based on their interaction scores.

## 3. Results

### 3.1. General Characteristics of the Patients with Endometrial Cancer and the Control Group of Patients

A total of 59 patients were included in the study. The tissue samples obtained from these patients had the following diagnoses: 17 of the tissue samples were NE, 3 endometrial polyps, 21 EH, and 18 had an EC diagnosis. The general and clinical features of the study population, classified according to their histopathological diagnosis, are shown in Table 1.

The mean and standard deviation for age (years) at diagnosis in the EP group of patients was 45.6 ± 14.18, in EH it was 45.47 ± 13.35, and in EC it was 56.22 ± 14.18. Among the group of patients with EC, 8 (44.4%) had systemic arterial hypertension (SAH). As for type 2 diabetes mellitus (T2DM), only 2 patients (11.1%) had it. The most frequent initial clinical manifestation in all the endometrial lesions was the abnormal uterine bleeding. For the group of patients with EC, 44.4% of the tumors had undergone metastasis.

### 3.2. Association of the Tissue Levels of miRNA-185-5p and miRNA-191-5p with Endometrial Cancer

Before conducting the statistical comparisons, it was essential to accurately establish the control group (calibrator) for its use in evaluating gene expression. This was to ascertain whether the tissue expression of the miRs of interest fluctuated in accordance with the stages of an NE. Consequently, the data related to the expression of miR-191-5p and miR-185-5p in normal NE were sub-categorized into proliferative and secretory subtypes. Subsequently, we compared the expression levels of these miRs between the subtypes (Table 2).

There was significant difference in miR-191-5p expression levels (*p* = 0.04) between proliferative and secretory endometrium subtypes, and a statistical trend for the miR-185-5p expression was also observed (*p* = 0.094). Consequently, NE was first included in the statistical data modeling as a single group, with subsequent analyses considering stratification into proliferative and secretory subtypes. Based on the above, and to evaluate the associations between the expression level of the miRs and EC, the patients and samples were stratified according to the current international clinical and pathological classifications, and the comparison of the tissue expression of each miR was compared among subgroups. The results of these comparisons are displayed in Table 2, and in Supplementary Figures S1 and S2.

**Figure 1:**
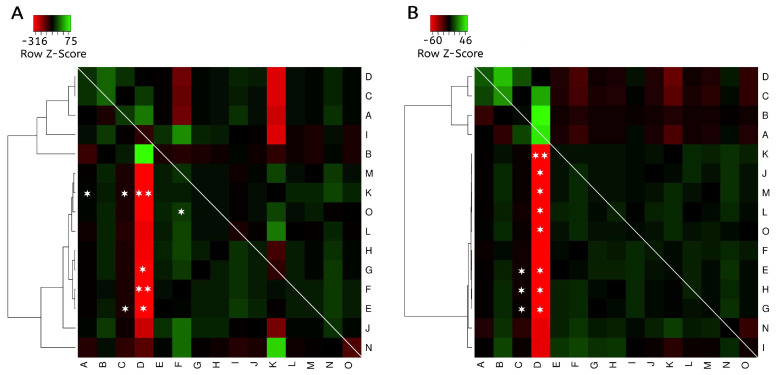
Supervised clustering analysis for hsa-miR-185 and hsa-191-5p in normal endometrial tissue and endometrial lesions. On the left of each heat map there is a tree diagram grouping those subgroups with similar expression profiles. (**A**) miRNA-185-5p. (**B**) miRNA-191-5p. * *p* ˂ 0.05, ** *p* < 0.09. Comparisons were carried out by using student’s *t* test. The basis of the comparison is row versus column. A, normal endometrium; B, proliferative endometrium; C, secretory endometrium; D, endometrial polyps; E, endometrial hyperplasia; F, endometrial hyperplasia with atypia; G, endometrial hyperplasia without atypia; H, endometrial cancer; I, Grade 1; J, Grade 2; K, Grade 3; L, FIGO I; M, FIGO III; N, myometrial invasion < 50%; O, myometrial invasion ≥ 50%.

Significant difference in the expression level of miR-185-5p was observed between groups of EC Type I and II, according the Bokhman Classification (Table 2). Supplementary Figure S1 displays the expression levels of the miRNA-185-5p (Figure S1A–D) and miRNA-191-5p (Figure S1E–H) among the different types of endometrial tissue. A difference in miR-185-5p expression was found between EP and EH (*p* < 0.05) and a trend toward significance with EC (*p* = 0.068). Likewise, a significant difference between these groups was found for miR-191-5p (*p* < 0.05). Considering NE tissue as a reference, the expression levels of both miRs were not differentially modulated in the EC, EH, or EP groups (*p* > 0.05). Supplementary Figure S2 displays a summary of the results of each pair of comparisons for the expression level for each miR among the subgroups of patients included in the study. With the aim of identifying clusters with similar tissue expression patterns of each miR, heat maps were generated in which each histological subtype was analyzed. For this analysis, the fold change (FC) was calculated and used during comparisons. The results are shown in Figure 1, where overexpression is represented in a green and underexpression in a red color.

In a general manner, two principal clades were identified for both miRs, grouping NE, PE, SE, and EPs, and EH and EC. However, for miR-185-5p (Figure 1A) EC grade 1 was also grouped, with no tumoral lesions. Considering the FC obtained for miR-185-5p, this miR was found to be underexpressed in EC grade 3 when compared to NE, SE, EHWA, and EC grade 1 (FC: −57.9 to −8.5, *p* ˂ 0.05). As for EH, it was found to be underexpressed compared with SE and EPs (FC: −54.1 to −9.9, *p* < 0.05). Likewise, underexpression was observed in EHWA compared with EPs (FC = −36.9, *p* ˂ 0.05). Overexpression with a significant difference was observed in those with myometrial invasion < 50% compared to EC grade 3 and EHA (FC: 23.4–40.3, *p* ˂ 0.05). On the other hand, an underexpression with a trend towards statistical significance was observed in grade 3 EC and EHA compared to EPs (FC: −315.7 to −183.1, *p* ˂ 0.09). As shown in Figure 1B, miR-191-5p was significantly underexpressed in EC, EH and EHWA when contrasted with SE and EPs (FC: −3.9 to −32.8, *p* ˂ 0.05). An underexpression with a trend toward statistical significance was also found in grade 3 EC compared with EPs (FC = −59.6, *p* ˂ 0.07). Overexpression of this miR was also observed in SE, EP and EC with myometrial invasion <50% when compared with PE (FC: 3.3–45.2, *p* ˂ 0.05). Considering the similar expression patterns identified for both miRs, Pearson correlation tests were carried out to identify the relationship between both miRs. The correlation for miR-185-5p and miR-191-5p in the NE showed a positive correlation coefficient of 0.953 (*p* = 9.13 × 10^−11^). For EH, a Pearson correlation coefficient between these miRs was 0.918 (*p* = 4.33 × 10^−9^), and in EC it was 0.800 (*p* = 0.00007).

To assess the impact of comorbidities such as hypertension and T2DM on miR expression, we classified the miR expression profiles of patients in each study group according to the presence or absence of these comorbidities. Unfortunately, as shown in Table 1, the number of patients with hypertension and/or T2DM in the NE, EP, and EH groups was small. Therefore, we evaluated the effect of hypertension only in the EC group. No significant differences were observed in miR-185-5p or miR-191-5p expression between EC patients with and without hypertension (*p* > 0.05).

### 3.3. Signaling Pathway Modulated by Hsa-miR-185-5p and Has-miR-191-5p

The web server DIANA-mirPath was used to identify target genes and cellular signaling pathways regulated by miRNA-185-5p and miR-191-5p. The results are summarized in Table 3. Three genes were found to be regulated by both miRs: SET domain containing 1B, histone lysine methyltransferase (*SETD1B*), tight junction protein 1 (*TJP1*), and Musashi RNA-binding protein 1 (*MSI1*). From the common genes, signaling pathways that could be regulated by both miRs were obtained, as shown in Table 3 [29]. Lysine degradation, Adherent unions, mRNA surveillance pathway and Gap Junctions, were among the significant signaling pathways in which these miRs and genes are involved (*p* ˂ 0.05).

## 4. Discussion

In this study, we conducted an expression analysis of miRs in non-tumoral and tumoral endometrial tissue to evaluate the usefulness of tissue expression of miR-185-5p and miR-191-5p as diagnostic markers of EC and their correlation with clinical and histopathological variables of the disease. MiR-185-5p and miR-191-5p showed a notable underexpression in advanced tumor grades. Although the involvement of these miRs in different lesions of endometrial tissue has not been studied, our results could be compared with other works with similar objectives in other pathological processes; for example, it has been proved that the deregulation of miR-185-5p promotes cellular changes associated with carcinogenesis and has epigenetics effects in other non-EC tumors. Yoon et al. described the fact that miR-185-5p was attenuated in gastric cancer and its endogenous induction modulated epigenetic processes through the downregulation of proteins such as DNA methyltransferase 1 (DNMT1) and enhancer of zeste homolog 2 (EZH2), which both produce methylations [30,31,32]. In hepatocellular cancer, miR-185-5p showed suppression of tumorigenesis through the blocking of the DNMT1/PTEN/Akt pathway [33]. *PTEN* (phosphatase and tensin homolog) is a tumoral suppressor gene and is inhibited by the methylation of DNMT1 during DNA replication, promoting carcinogenesis [34,35]. Previous research has described a downregulation of miR-185-5p in breast cancer, where the miR-185-5p expression was influenced by vascular endothelial growth factor A (VEGFA), a key molecule involved in the formation of blood vessels. VEGFA is essential for initiating the development of immature vessels within tumors, which is a critical step in tumor angiogenesis [36,37,38]. Similarly, there is evidence of the importance of miR-191-5p in cancer. It is known that miR-191 can inhibit TNF-α-induced apoptosis in EC cell lines by downregulating *DAPK1*, which is a positive mediator of programmed cell death [22]. In colorectal cancer, miR-191-5p acts as a tumor suppressor because its endogenous induction inhibits cell proliferation and invasion through two molecular targets: special AT-rich sequence-binding protein 1 (SATB1) and tripartite motif-containing 14 (TRIM14) [39].

**Figure 2:**
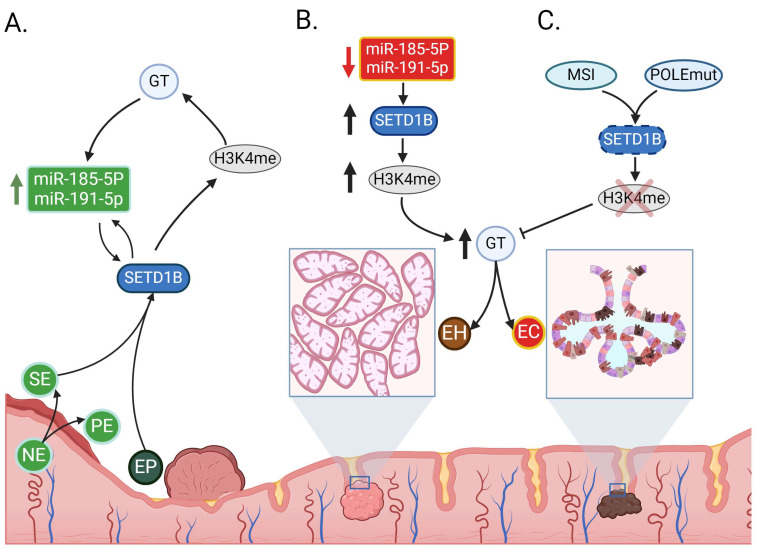
Proposed signaling pathways between miRs of interest with *SETD1B* in different types of endometrial tissues. (**A**) Functional *SETD1B* gene promotes histone H3K4 methylation, which regulates GT and maintains cellular control; the up-regulation of both miRs could regulate the expression level of *SETD1B* by negative feedback. In the case of PE, where there is a high rate of cell proliferation and thus an increase in GT, it is inferred that there will be an increase in *SETD1B* activity, and as a consequence, there will be a decrease in miR185-5p and miR-191-5p levels, since, otherwise, these miRs could inhibit *SETD1B* expression and thus hinder cell proliferation in PE. In those tissues characterized by greater cell quiescence, the opposite phenomenon is observed, and such is the case of the SE and EP, where we observed an overexpression of these miRs with the aim of downregulating *SETD1B* expression and thus the activation of transcription. (**B**) Downregulation of miRNAs overexpresses *SETD1B*, generates an increase in H3K4 methylation and over-activates gene transcription, upregulating oncogenes that favor endometrial carcinogenesis and endometrial invasion. (**C**) Deletions in the *MSI* reading frame and POLEmut mutations generate a non-functional *SETD1B* unable to methylate H3K4, which suppresses GT activation and downregulates tumor suppressor genes, initiating a vicious cycle that favors endometrial carcinogenesis. EP, Endometrial Polip; NE, Normal Endometrium; PE, proliferative endometrium; SE, secretory endometrium; EH, endometrial hyperplasia; EC, endometrial cancer; *SETD1B*, SET domain containing 1B, Histone Lysine Methyltransferase; H3K4me, Histone H3 lysine K4 methylation; GT, gene transcription; MSI, microsatellite instability; POLEmut, DNA polymerase epsilon mutated. The blue square indicates an amplified section of the endometrial tissue. The red cross indicates “unable to methylate”.

Although there is not a study that has investigated the functional mechanism of miR-185-5p and miR-191-5p and their molecular targets in EC, our in silico analysis show that there were three genes potentially modulated by these miRNAs: *SETD1B*, *TJP1* and *MSI1*. *SETD1B* (histone methyltransferase KMT2 family) is a gene involved in chromatin remodeling through histone methylation [40]. *SETD1B* takes part in methylation of the histone H3 tail at lysine 4 (H3K4), an epigenetic mechanism that regulates transcription activation [41]; changes in the degree of DNA methylation, chromatin structure, and post-translational modifications of histones may lead to the development of cancer [42]. The up-regulation of both miRs could regulate the expression level of *SETD1B* by negative feedback. It is understood that underexpression of both miRs leads to an overexpression of *SETD1B*, and thus, an increase in transcriptional activation leading to overexpression of oncogenes, which is necessary for a tumor or hyperplastic cell. Also, deletions with reading frame shift and nonsense mutations by *MSI* and *POLE*, respectively [43,44] could generate a non-functional *SETD1B*, which inactivates *H3K4* methylation and gene transcription, leading to a downregulation of tumor suppressor genes, and promoting carcinogenesis. Figure 2 shows a summary of the possible signaling pathways between the miRs of interest and the *SETD1B* in endometrial tissues. It is likely these miRs function as epi-miRs in EC, which are distinguished by their ability to directly or indirectly regulate the expression of several epigenetic regulators such as DNMTs and HDACs [9,10].

**Figure 3:**
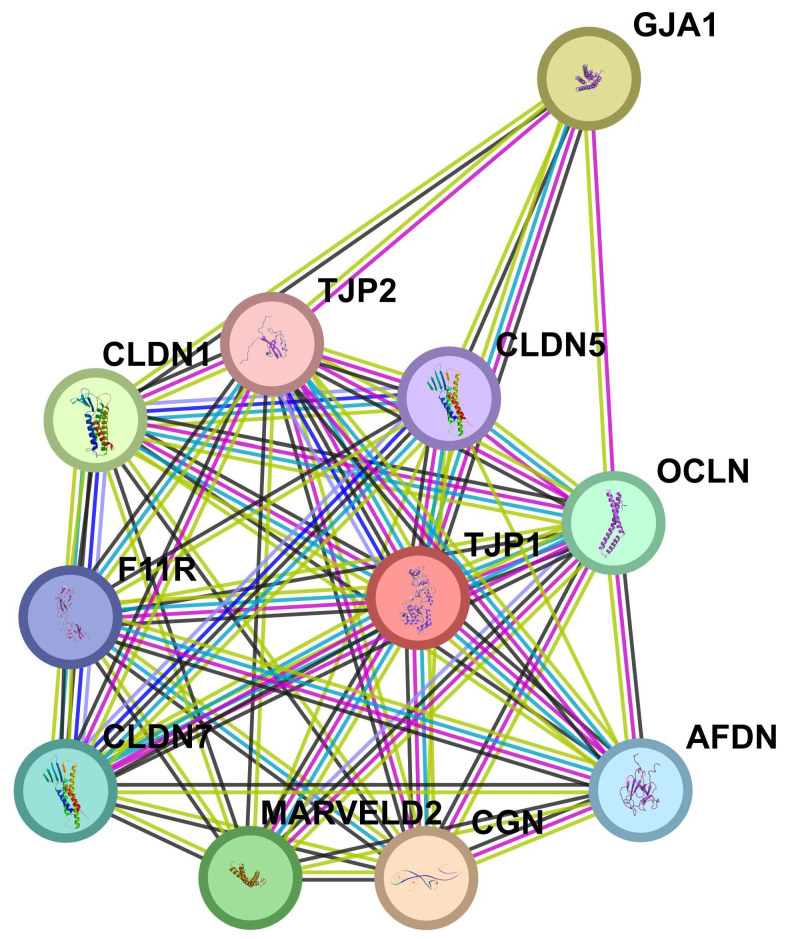
TJP1 Interactome. TPJ1 is a protein involved in the binding of tight junction (TJ) transmembrane proteins, such as claudins (CLDNs) and junctional adhesion molecules and occludin (OCLN), to the actin cytoskeleton. The tight junction limits the movement of substances through the paracellular space and between the apical and basolateral plasma membrane domains of epithelial and endothelial cells. Source: retrieved from STRING database (https://string-db.org/, accessed on 21 June 2024). TJP1, Tight Junction Protein 1; CGN, Cingulin; GJA1, Gap Junction Protein Alpha 1; CLDN1, Claudin 1; MARVELD2, MARVEL Domain Containing 2; OCLN, Occludin; CLDN7, Claudin 7; AFDN, Afadin; F11R, Junctional Adhesion Molecule A; CLDN5, Claudin 5; TJP2, Tight Junction Protein ZO-2.

TJP1 is a peripheral cellular protein, a member of the membrane-associated guanylate kinase (MAGUK) family. It interacts with occludins and claudins (Figure 3) and binds tight junction components to the actin cytoskeleton through its N-terminal region [45] to maintain the integrity of epithelial [46] and non-epithelial tight junctions [47]. TJP1 acts in two signaling pathways related to adherent unions and gap junctions [29] and contributes to cell proliferation in leiomyosarcoma by favoring cell–cell aggregation and cytokine-mediated communication in the tumor microenvironment [48]. TJP1 also promotes angiogenesis in tumor progression by moving from the membrane to the cytoplasm in A549 cells after TGF-β treatment. In addition, it can accelerate tumor angiogenesis by regulating the NFkB C-X-C motif chemokine ligand 8 (CXCL8) axis [49]. TJP1 may play a role in cancer progression, since the delocalization of this protein into cytoplasmic or nuclear compartments confers invasive and pro-tumorigenic functions [50]. For miR-185-5p, in which higher underexpression was observed among grade 3 EC compared to grade 1, it has been found that downregulation of *TJP1* attenuates cell–cell aggregation and anchorage-independent growth in tumor cells in vitro [51], suggesting that overexpression of *TJP1* (in advanced tumor grades) could stabilize tumor cells within connective tissue during tumor formation. On the other hand, and in the cell physiology context, *MSI1* acts in the mRNA surveillance signaling pathway. MSI1 is an intracellular protein that binds RNA and is involved in stem cell renewal, differentiation, and maintenance of pluripotency; it localizes mainly to the cell cytoplasm and nucleus [52]. In EH and EC tissues, MSI is significantly overexpressed in the former tissues compared with NE [53]. MSI1 is mainly localized in the cytoplasm of glandular cells in EC compared with the other tissues, where it is mainly located in the endometrial stroma [53]. According to the above, the significant underexpression of miR-191-5p in PE compared to SE observed in our study is in agreement with the results of Gotte et al. [54], which found a significant overexpression of *MSI1* in PE compared to SE (FC = 4, *p* ˂ 0.05). Other studies have demonstrated a significant elevation in *MSI1* mRNA expression of CEE tissue, with levels being 2.8 times higher compared to NE, which indicated a marked difference (*p* = 0.0009) [55]. Additionally, it has been described that the silencing of *MSI1* in EC in vitro leads to a notable reduction in both cell proliferation and radioresistance (*p* < 0.05) [56]. *MSI1* suppression led to downregulation of telomerase, the DNA-dependent protein kinase, the Notch pathway, and overexpression of the cyclin-dependent kinase inhibitor p21, the last of which was identified as a key mediator of antiproliferative signaling related to MSI1 suppression. Based on the above findings, we can infer that the miR-185-5p and miR-191-5p, by downregulating *MSI1*, may cause decreased EC proliferation and loss of radioresistance, suggesting therapeutic potential.

In spite of our study being the first to offer a comprehensive analysis of miR-185-5p and miR-191-5p expression across a continuum of endometrial conditions, ranging from NE to various stages of malignancy including benign lesions, hyperplasia, and high-grade EC, some study limitations should be recognized. These limitations are notably related to its retrospective nature. Due to this, we were unable to access detailed treatment data, as patients were managed at a different medical facility. This lack of access to patient treatment information presents a challenge in fully understanding the interplay between miRNA expression and therapeutic outcomes. Additionally, our study did not encompass the molecular characterization and classification of the tumor, a step that is crucial for confirming the roles of miR-185-5p and miR-191-5p in the pathogenesis of EC. Given these gaps, further research is warranted to evaluate the involvement of these miRs and their correlations with disease progression. Prospective studies with comprehensive molecular profiling are essential to validate our findings and to potentially integrate these miRs into a clinical setting for diagnostic or prognostic utility. However, the significant underexpression of miR-185-5p and miR-191-5p in EC tissues compared to non-tumor tissues identified in our study not only enhance our understanding of the complex molecular landscape of EC but also underscores the importance of miRNAs as potential diagnostic and prognostic tools of this disease.

## 5. Conclusions

The tissue expression levels of miR-185-5p and miR-191-5p were significantly reduced in EC, particularly in high-grade tumors, when compared with benign endometrial tissue, including EPs. This underexpression correlated with advanced histopathological features of the disease, suggesting that these miRNAs may serve as molecular biomarkers of EC. Notably, EPs presented a unique miRNA expression profile, distinct from other endometrial lesions. In relation to their molecular targets, our findings propose that miR-185-5p and miR-191-5p may act as tumor suppressors and could play a role as epigenetic modulators (epi-miRs) in EC.

## Figures and Tables

**Table 1:** General and clinical characteristics of the study population.

Feature	Study Group			
	Normal Endometrium (n = 17)	Endometrial Polyps (n = 3)	Endometrial Hyperplasia (n = 21)	Endometrial Cancer (n = 18)
Age at diagnosis (years)				
<50 n (%)	16 (94.1)	2 (66.6)	17 (80.9)	5 (27.7)
50–70 n (%)	1 (5.9)	1 (33.3)	3 (14.2)	9 (50.0)
>70 n (%)	0 (0)	0 (0)	1 (4.7)	4 (22.2)
Initial clinical manifestation				
Abnormal uterine bleeding n (%)	10 (58.8)	3 (100)	21 (100)	17 (94.4)
Asymptomatic n (%)	7 (41.1)	0 (0)	0 (0)	0 (0)
Unknown n (%)	0 (0)	0 (0)	0 (0)	1 (5.6)
Comorbidities				
Systemic arterial hypertension n (%)	1 (5.8)	0 (0)	2 (9.5)	8 (44.4)
Type 2 diabetes mellitus n (%)	1 (5.8)	0 (0)	2 (9.5)	2 (11.1)
Medical treatment				
Chemotherapy n (%)	NA	NA	NA	5 (27.7)
Radiotherapy n (%)	NA	NA	NA	7 (38.8)
Metastasis n (%)	NA	NA	NA	8 (44.4)

Data are presented in frequency and percentage. NA, not applicable.

**Table 2:** Tissue expression of miR-185-5p and miR-191-5p in different endometrial tissue subgroups. Differences in miR-191-5p expression were found between proliferative and secretory endometrium.

Tissue Sample or Classification	n	Tissue Expression (Log 2 of Expression Level)			
		miR-185-5p	*p*-Value	miR-191-5p	*p*-Value
Normal endometrium	17	−0.6 ± 4.573	NA	−0.595 ± 2.625	NA
Proliferative endometrium	7	−2.824 ± 3.092	0.094 ^a^	−2.126 ± 1.127	0.04 *^,a^
Secretory endometrium	10	0.956 ± 4.926		0.477 ± 2.881	
Endometrial polyps	3	3.402 ± 6.968	NA	3.371 ± 4.974	NA
Endometrial hyperplasia	21	−2.355 ± 2.765	NA	−1.665 ± 1.274	NA
With atypia	5	−4.115 ± 3.447	0.104 ^a^	−2.812 ± 1.923	0.117 ^b^
Without atypia	16	−1.805 ± 2.381		−1.579 ± 1.013	
Endometrial cancer	18	−1.524 ± 3.597	NA	−1.587 ± 1.679	NA
Bokhman Classification					
Type I	13	−0.367 ± 3.535	0.023 *^,a^	−1.316 ± 1.808	0.282 ^a^
Type II	5	−4.530 ± 1.367		−2.293 ± 1.144	
Tumor grade (endometrioid)					
G1	5	0.323 ± 3.334	0.073 ^c^	−0.619 ± 2.374	0.239 ^c^
G2	9	−1.049 ± 3.642		−1.708 ± 1.269	
G3	4	−4.901 ± 1.256		−2.525 ± 1.177	
FIGO Stage					
I	10	−1.301 ± 3.628	0.628 ^c^	−1.502 ± 2.032	0.765 ^c^
III	7	−2.009 ± 4.041		−1.742 ± 1.320	
IV	1	−0.349 ± 0.000		−1.357 ± 0.000	
Myometrial invasion					
<50%	4	0.212 ± 1.293	0.137 ^b^	−0.412 ± 1.287	0.115 ^a^
≥50%	14	−3.458 ± 3.876		−1.923 ± 1.661	
Age in years (EC)					
≤50	5	0.807 ± 4.708	0.088 ^a^	−0.75 ± 2.067	0.198 ^a^
>50	13	−2.42 ± 2.794		−1.909 ± 1.471	

Data are represented as mean ± standard deviation. NA, not applicable; EC, endometrial cancer; FIGO, International Federation of Obstetrics and Gynecology. * *p* < 0.05, ^a^ Student’s *t*-test, ^b^ Mann– Whitney U test, ^c^ One-way ANOVA test.

**Table 3:** Gene intersection for miRNA-185-5p and miRNA-191-5p [29].

KEGG Signaling Pathway	*p*-Value	Target Genes	miRs Involved	Target Gene	Ensembl ID
Lysine degradation	3.83 × 10^−5^	1	2	*SETD1B*	ENSG00000139718
Adherent unions	0.0024	1	2	*TJP1*	ENSG00000104067
mRNA surveillance pathway	0.0368	1	2	*MSI1*	ENSG00000135097
Gap Junctions	0.0368	1	2	*TJP1*	ENSG00000104067
Infection by *Vibrio cholerae*	0.0368	1	2		
Epithelial cell signaling in *H. pylori* infection	0.0368	1	2		
Salmonella infection	0.0368	1	2		

Ensembl ID, gene identifier in Ensembl database; KEGG, Kyoto Encyclopedia of Genes and Genomes; mRNA, messenger ribonucleic acid; *SETD1B*, SET domain containing 1B, Histone Lysine Methyltransferase; *TJP1*, Tight Junction Protein 1; and *MSI1*, Musashi RNA Binding Protein 1.

## Data Availability

The data generated in the present study may be requested from the corresponding authors.

## Supplementary Materials

The following supporting information can be downloaded at: https://www.mdpi.com/article/10.3390/cells13131099/s1, Figure S1: miRNA-185-5p (A–D) and miRNA-191-5p (E–H) expression levels between healthy endometrial tissue, precancerous lesions, and EC.; Figure S2: Summary of comparisons of the hsa-miR-185-5p and has-miR-191-5p expression levels between subgroups of study participants.
