# Supplementary material for: Comparative Assessment of miR-185-5p and miR-191-5p Expression: From Normal Endometrium to High-Grade Endometrial Cancer
Source: Cells. 2024 Jun 25;13(13):1099. doi: 10.3390/cells13131099 (PMC11240595; doi:10.3390/cells13131099)
Supplement: Supplementary file 1 [file cells-13-01099-s001.zip › cells-3042235-supplementary.pdf]

Supplementary File

Comparative Assessment of miR-185-5p and miR-191-5p Expression: From Normal Endometrium to High-Grade Endometrial Cancer

Sergio Antonio Oropeza-de Lara [1], Idalia Garza-Veloz [1,*], Bertha Berthaud-González [2], Tania Guillermina Tirado-Navarro [1], Reinaldo Gurrola-Carlos [1], Bernardo Bonilla-Rocha [1], Ivan Delgado-Enciso [3,4] and Margarita L. Martinez-Fierro [1,*]

[1] Molecular Medicine Laboratory, Academic Unit of Human Medicine and Health Sciences, Universidad Autonoma de Zacatecas, Carretera Zacatecas-Guadalajara Km 6 Ejido la Escondida, Zacatecas 98160, Mexico; sergioantoniooropeza@gmail.com (S.A.O.-d.L.); dra.tania.tirado@gmail.com (T.G.T.-N.); reinaldogc@gmail.com (R.G.-C.); bern.ave96@gmail.com (B.B.-R.).

[2] Hospital General “Luz González Cosío”, Circuito el Orito, Cd. Administrativa, Zacatecas 98160, Mexico; bberthaud@hotmail.com

[3] Department of Molecular Medicine, School of Medicine, University of Colima, Av. Universidad No. 333, Las Viboras, Colima 28040, Mexico; ivan_delgado_enciso@ucol.mx

[4] Department of Research, Colima Cancerology State Institute, IMSS-Bienestar Colima, Colima 28085, Mexico

* Correspondence: idaliagv@uaz.edu.mx (I.G.-V.); margaritamf@uaz.edu.mx (M.L.M.-F.); Tel.: +52-492-9256690 (ext. 2109) (I.G.-V.); +52-492-9256690 (ext. 4535) (M.L.M.-F.)

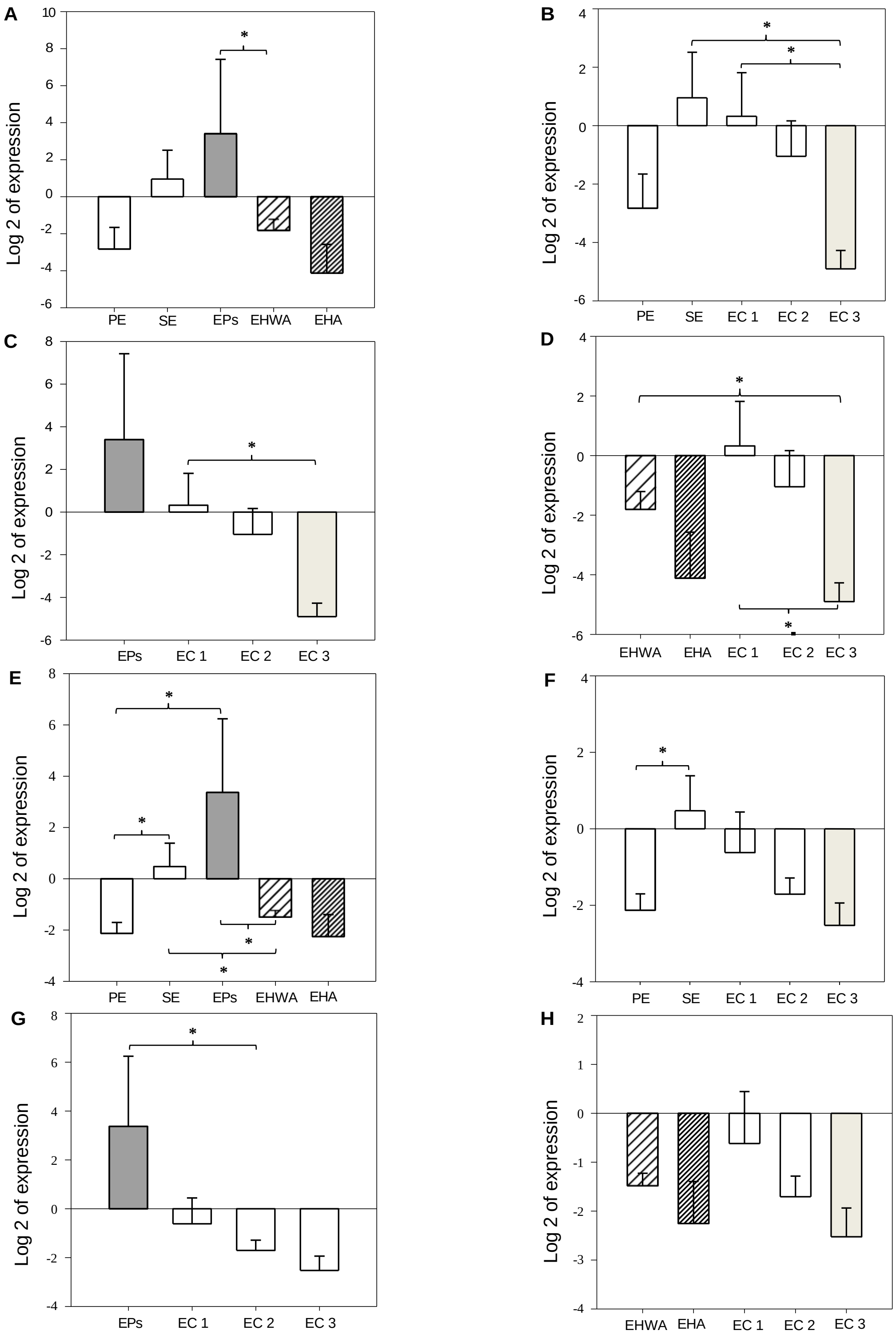

Supplementary Figure S1. miRNA-185-5p (A-D) and miRNA-191-5p (E-H) expression levels between healthy endometrial tissue, precancerous lesions, and EC. (A/E) Expression levels in healthy endometrial tissue and precancerous lesions. (B/F) Expression levels in healthy endometrial tissue and EC. (C/G) Expression levels in EPs and EC grades. (D/H) Expression levels in precancerous lesions and EC. * $p < 0.05$, student's t test. NE, Normal endometrium; SE, Secretory endometrium; PE, Proliferative endometrium; Eps, Endometrial polyps; EHWA, Endometrial hyperplasia without atypia; HEA, Endometrial hyperplasia with atypia; EC, Endometrial cancer.

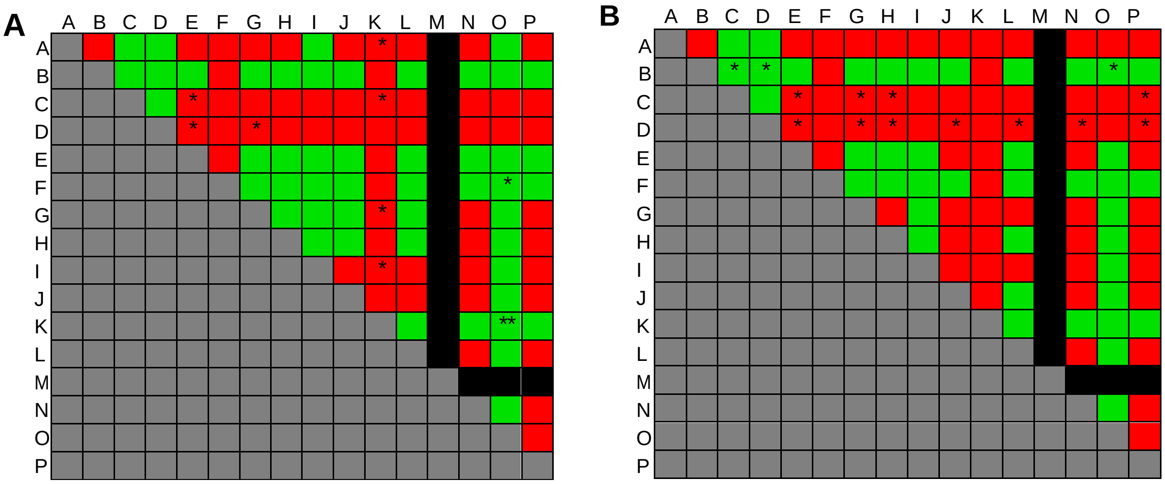

Supplementary Figure S2. Summary of comparisons of the hsa-miR-185-5p and has-miR-191-5p expression levels between subgroups of study participants. (A) displays the results of each pair of comparison of miRNA-185-5p expression level between each subgroup. There was an underexpression statistically significant in grade 3 EC compared to other non-tumor tissues, e.g. NE, SE and ENWA. For miRNA-191-5p (B), several subgroups of precancerous and tumor lesions show underexpression compared to EPs. * $p < 0.05$, ** $p \leq 0.001$. Comparisons were obtained using student's t-test. The green color represents tissue overexpression and in red is represented the under-expression. The black color represents samples of the lesion were not identified. The comparison is in the direction of the column versus the row. A, Normal endometrium; B, Proliferative endometrium; C, Secretory endometrium; D, Endometrial polyps; E, Endometrial hyperplasia; F, Endometrial hyperplasia with atypia; G, Endometrial hyperplasia without atypia; H, Endometrial cancer; I, Grade 1; J, Grade 2; K, Grade 3; L, FIGO I; M, FIGO II; N, FIGO III; O, Myometrial invasion < 50%; P, Myometrial invasion ≥ 50%; EC, Endometrial cancer; NE, Normal endometrium; SE, Secretory endometrium; ENWA, Endometrial hyperplasia without atypia; EP, Endometrial polyps.
